# Population genetic diversity in the annual breeding area of the *Spodoptera frugiperda* in China

**DOI:** 10.1038/s41598-026-46482-1

**Published:** 2026-04-02

**Authors:** J. R. Lin, J. Zhang, Y. Zou, Z. W. Wu

**Affiliations:** College of Coastal Agricultural Sciences, Guangdong Ocean University, Zhanjiang, 524088 Guangdong China

**Keywords:** Fall armyworm (FAW), Genetic diversity, Haplotype, Genetic differentiation, Invasion biology, Evolutionary genetics, Population genetics, Invasive species

## Abstract

The fall armyworm (*Spodoptera frugiperda*), a globally invasive pest, has established persistent populations in China since its invasion in late 2018, posing a continuous threat to agriculture due to its potential for rapid adaptation and insecticide resistance evolution. To elucidate the genetic underpinnings of its invasion success in China’s annual breeding area, this study systematically analyzed the genetic diversity, differentiation, and population structure of fall armyworm (FAW) populations across four provinces (Yunnan, Guangdong, Guangxi, and Hainan) using a dual-marker system combining mitochondrial *COI* and nuclear *Tpi* genes. Our analyses revealed significantly reduced genetic diversity (haplotype diversity: 0.47–0.56; nucleotide diversity: 0.007–0.009) in Chinese populations compared to putative source populations in India (0.942 and 0.014, respectively), indicating a strong genetic bottleneck effect. All individuals were identified as the corn-strain, with 22.7% classified as heterozygous corn-strain based on *Tpi* genotyping. Population differentiation analysis highlighted the Himalayas as a substantial barrier (*F*_*st*_ = 0.294 between Yunnan and India), while the South China Sea monsoon facilitated genetic fusion between Guangdong and Hainan (*F*_*st*_ = -0.033). Guangxi emerged as a gene flow hub, exhibiting higher haplotype diversity likely due to continuous influx from the China-Vietnam border. Furthermore, significantly positive Tajima’s D values (3.08–3.94) in Chinese populations, coupled with field-observed population fluctuations, support an invasion model characterized by rapid expansion from a limited number of founders. This study provides critical insights into the population genetic dynamics of fall armyworm in its newly invaded range, which are essential for forecasting its spread and formulating sustainable management strategies.

**Supplementary Information:**

The online version contains supplementary material available at 10.1038/s41598-026-46482-1.

## Introduction

The fall armyworm (FAW), *Spodoptera frugiperda*, is a highly destructive migratory pest native to the Americas^1,2^ that has achieved global notoriety following its recent invasions across Asia and Africa^3–5^. Since its invasion of Africa in 2016^6^ and subsequent spread to Asia, it has posed a severe and persistent threat to global food security. FAW was first detected in Yunnan Province, China, in December 2018^7^ and rapidly colonized 26 provinces within a year^7,8^, establishing permanent populations in its annual breeding areas across southern China. Understanding the genetic diversity and population structure of these invasive populations is crucial for developing effective monitoring and management strategies, given their remarkable adaptability and potential for evolving insecticide resistance ^9^.

Previous studies on FAW in China have yielded important yet partially inconsistent findings. Some reports identified all early invaders in Yunnan as the corn-strain^15^, while others detected rice-strain haplotypes in central and southern provinces^16^. Genetic diversity analyses have generally indicated lower diversity in Chinese FAW populations compared to native American or South Asian populations, suggesting founder effects post-invasion^9,17^. However, systematic assessments of genetic variation across China’s annual breeding area—encompassing key provinces such as Guangdong, Guangxi, Hainan, and Yunnan—remain limited. Furthermore, the patterns of gene flow, genetic differentiation, and the potential source–sink relationships between Chinese populations and those in neighboring regions (e.g., India, South Korea) are still poorly resolved^17,18^.

Molecular markers are indispensable tools for deciphering the genetic architecture and migration patterns of invasive insects. The mitochondrial cytochrome oxidase I (*COI*) gene serves as a standard barcode for species identification and population genetic studies due to its high mutation rate and maternal inheritance^10,11^. Additionally, the Z-linked triosephosphate isomerase (*Tpi*) gene provides a complementary nuclear marker that reliably distinguishes between the host-associated corn- and rice-strains of FAW, offering insights into host adaptation and population origins^12,13^. The combined use of *COI* and *Tpi* markers can yield a more robust and comprehensive understanding of FAW invasion dynamics^14^.

To address these knowledge gaps, this study aimed to (1) comprehensively assess the genetic diversity and population structure of FAW within China’s annual breeding area using both *COI* and *Tpi* markers; (2) compare these patterns with populations from potential source regions (India) and neighboring invaded areas (South Korea); and (3) elucidate the demographic history and possible migration routes that have shaped the current genetic landscape of FAW in China. The findings are expected to enhance our understanding of FAW’s adaptive evolution in newly invaded habitats and provide a genetic basis for targeted regional management strategies.

## Materials and methods

### Sample collection

**Fig. 1:**
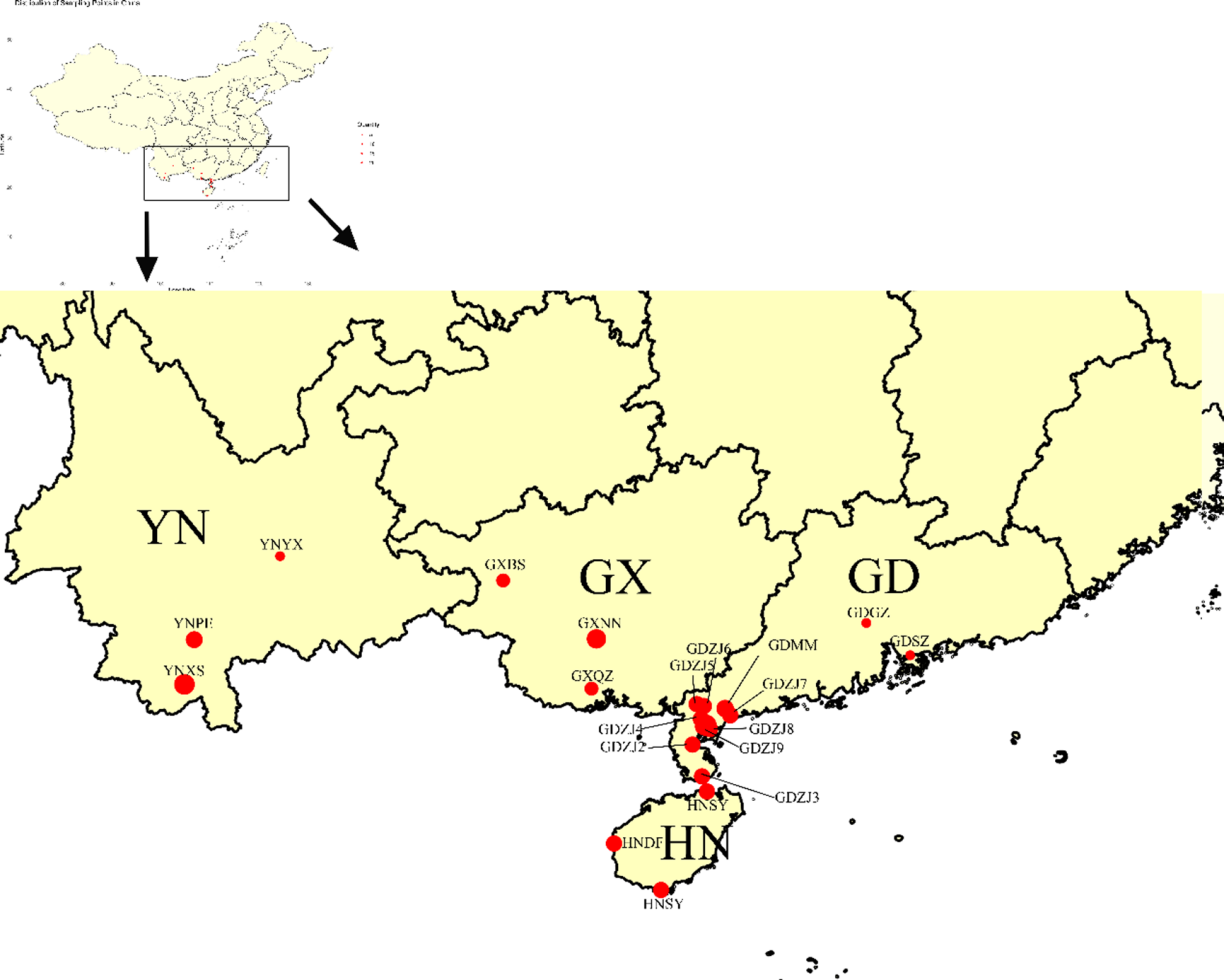
Sampling locations of fall armyworm (FAW) populations in China. Solid red circles are sampling locations for this study. *Note*: The sampling map was created using: R programming language (v4.3.2; https://www.r-project.org/) sf package (v1.0–16; https://r-spatial.github.io/sf/) for spatial data processing ggplot2 package (v3.5.0; https://ggplot2.tidyverse.org) for visualization GDAL library (v3.8.2; https://gdal.org) as underlying spatial engine.

A total of 123 fall armyworm (FAW) larvae (3rd-5th instar) were collected from 21 geographic locations across four provinces (Guangdong, GD; Guangxi, GX; Hainan, HN; Yunnan, YN) within China’s annual breeding area during 2023 (Table 1, Fig. 1). All samples were preserved in absolute ethanol at − 80°C until DNA extraction.

**Table 1:** Fall armyworm (FAW) samples collection information.

Geographic region	Sampling locations	Sampling Code	Sampling date	No.of individual	Longitude Latitude	Accession numbers
Guangxi(GX)	Nanning	GXNN	2023–03-15	5	108.23°E, 22.61°N	PQ129486-PQ129490
	Qinzhou	GXQZ	2023–03-16	5	108.59°E, 21.93°N	PQ129908-PQ129912
	Baise	GXBS	2023–03-17	5	106.72°E, 23.59°N	PQ129897-PQ129901
Yunnan(YN)	Yuxi	YNYX	2023–03-18	5	102.57°E, 24.29°N	PQ129943-PQ129947
	Puer	YNPE	2023–03-19	6	101.05°E, 23.05°N	PQ129921-PQ129926
	Xishuangbanna	YNXS	2023–03-19	10	100.77°E, 21.98°N	PQ129927-PQ129936
Guangdong(GD)	Potou, Zhanjiang	GDZJ1	2023–03-04	5	110.46°E, 21.24°N	PQ057544-PQ057548
	Leizhou, Zhanjiang	GDZJ2	2023–02-22	6	110.10°E, 20.91°N	PQ129857-PQ129862
	Xuwen, Zhanjiang	GDZJ3	2023–02-22	6	110.18°E, 20.33°N	PQ129316-PQ129321
	Suixi, Zhanjiang	GDZJ4	2023–02-23	6	110.25°E, 21.38°N	PQ129869-PQ129874
	Lianjiang, Zhanjiang	GDZJ5	2023–02-23	6	110.29°E, 21.61°N	PQ129863-PQ129868
	Kaifaqu, Zhanjiang	GDZJ6	2023–02-21	5	110.16°E, 21.65°N	PQ129875-PQ129879
	Wuchuan, Zhanjiang	GDZJ7	2023–02-23	6	110.78°E, 21.44°N	PQ129880-PQ129885
	Xiashan, Zhanjiang	GDZJ8	2023–02-21	5	110.40°E, 21.19°N	PQ129886-PQ129890
	Mazhang, Zhanjiang	GDZJ9	2023–03-14	6	110.33°E, 21.26°N	PQ129891-PQ129896
	Huazhou, Maoming	GDMM	2023–03-02	6	110.64°E, 21.66°N	PQ129286-PQ129291
	Shenzhen	GDSZ	2023–03-04	5	114.06°E, 22.54°N	PQ129852-PQ129856
	Guangzhou	GDGZ	2023–03-04	5	113.26°E, 23.13°N	PQ056730-PQ056734
Hainan(HN)	Sanya	HNSY	2023–03-03	6	109.51°E, 18.25°N	PQ129937-PQ129942
	Dongfang	HNDF	2023–03-02	6	108.65°E, 19.10°N	PQ129902-PQ129907
	Haikou	HNHK	2023–04-08	8	110.20°E, 20.05°N	PQ129913-PQ129920

### DNA extraction, PCR amplification, and sequencing

Genomic DNA was extracted from each individual larva using the TIANGEN DNA kit (TIANGEN Biotech, Beijing) according to the manufacturer’s instructions. The mitochondrial *COI* gene fragment was amplified using primers Sf-*COI*-F/Sf-*COI*-R^10^, and the nuclear *Tpi* gene fragment was amplified using primers Sf-*Tpi*-F/Sf-*Tpi*-R^19^. PCR reactions (50 μL) contained 25 μL of PCR MIX, 2 μL of each primer (20 pmol/μL), 2 μL of DNA template (50 ng/μL), and 19 μL of sterile water. The thermocycling profile was: initial denaturation at 94°C for 5 min; 35 cycles of denaturation at 94°C for 30 s, annealing at 55°C for 30 s, and extension at 72°C for 60 s; followed by a final extension at 72°C for 30 min^9^. PCR products were verified by 1% agarose gel electrophoresis and sent to Sangon Biotech (Shanghai, China) for bidirectional Sanger sequencing.

### Strain identification using the *Tpi* gene

The obtained *Tpi* sequences were aligned with reference sequences for the corn-strain and rice-strain of *Spodoptera frugiperda* retrieved from GenBank, using ClustalX software^20^. Strain identity for each sample was determined based on diagnostic nucleotide sites as defined in previous studies^21^.

### Sequence alignment and dataset compilation

Chromatograms were checked, and *COI* sequences were assembled and manually edited using SeqMan 7.1.0 (DNASTAR, Madison, WI, USA). To enable cross-regional comparison, additional *COI* sequences of fall armyworm (FAW) populations from India and South Korea were downloaded from the NCBI GenBank database. All *COI* sequences (newly generated and publicly available) were aligned using the ClustalW algorithm implemented in MEGA 11^22^, with a final trimmed length of 646 bp for consistent analysis.

### Genetic polymorphism and diversity analysis

Genetic diversity indices—including the number of polymorphic sites, number of haplotypes, haplotype diversity (Hd), and nucleotide diversity (π)—were calculated for each geographic population using DnaSP v5.0^23^. Neutrality tests (Tajima’s D, Fu’s Fs, Fu and Li’s D* and F*) were also performed using the same software to infer demographic history.

### Genetic differentiation and gene flow analysis

Genetic differentiation among populations was quantified by pairwise *F*_*st*_ values, and gene flow was estimated as Nm (where Nm = ∞ indicates infinite gene flow), using Arlequin 3.5^24^. The overall genetic structure was assessed by Analysis of Molecular Variance (AMOVA) implemented in the same software. Additionally, the effective number of migrants per generation was estimated using MIGRATE-N v3.2.16^25^.

### Phylogenetic and haplotype network construction

A neighbor-joining (NJ) phylogenetic tree based on *COI* haplotypes was constructed with 1,000 bootstrap replicates in MEGA 11^26^. A haplotype network was generated using the TCS method^27^ in PopART v1.7^28^ to visualize the evolutionary relationships among haplotypes.

### Demographic history analysis

Historical population dynamics were investigated using two complementary approaches. First, the mismatch distribution of pairwise genetic differences was calculated and tested for goodness-of-fit to a model of sudden population expansion using Arlequin 3.5^24^. Second, the same software was used to compute the sum of squared deviations (SSD) and the Harpending’s raggedness index (*r*) to further evaluate the expansion model.

## Results

### Strain Identification via the *Tpi* Gene

**Fig. 2:**
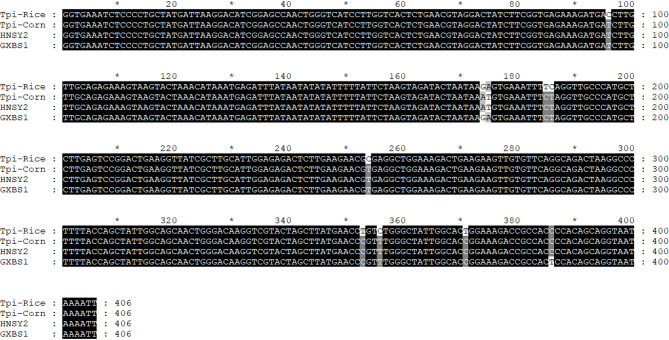
Identification of subtypes of S. *frugiperda* based on *Tpi* gene fragment.

Strain identity was determined by aligning the obtained *Tpi* gene sequences with reference sequences for the corn-strain and rice-strain of *Spodoptera frugiperda* ^21^. Comparative sequence analysis revealed that all 123 individuals from the annual breeding area exhibited the genetic signature of the corn-strain. A representative sequence alignment (e.g., sample HNSY2) showed 100% identity with the canonical corn-strain sequence, while differing from the rice-strain at nine diagnostic nucleotide positions (Fig. 2). Key differentiating sites included C/T at position 96, GA/AT at positions 174–175, and TC/CT at positions 186–187, among others.

Furthermore, 28 samples (22.8%) displayed a heterozygous pattern at positions 175–176, matching the rice-strain (GA) at this site but aligning with the corn-strain at all other diagnostic loci. These were consistently classified as heterozygous corn-strain. These results confirm that the invasive fall armyworm (FAW) populations in China’s annual breeding area are predominantly, if not exclusively, of corn-strain origin, with a notable proportion exhibiting heterozygosity at a specific site within the *Tpi* gene.

### Genetic diversity

The target *COI* gene region (814 bp) was successfully amplified and sequenced from all 123 fall armyworm (FAW) specimens collected in this study. The resulting nucleotide sequences were deposited in the GenBank database under accession numbers PQ129486–PQ129920 (Table 1). Sequence analysis identified a total of 36 polymorphic sites, which defined 12 distinct haplotypes, designated Hap1 to Hap12.

**Fig. 3:**
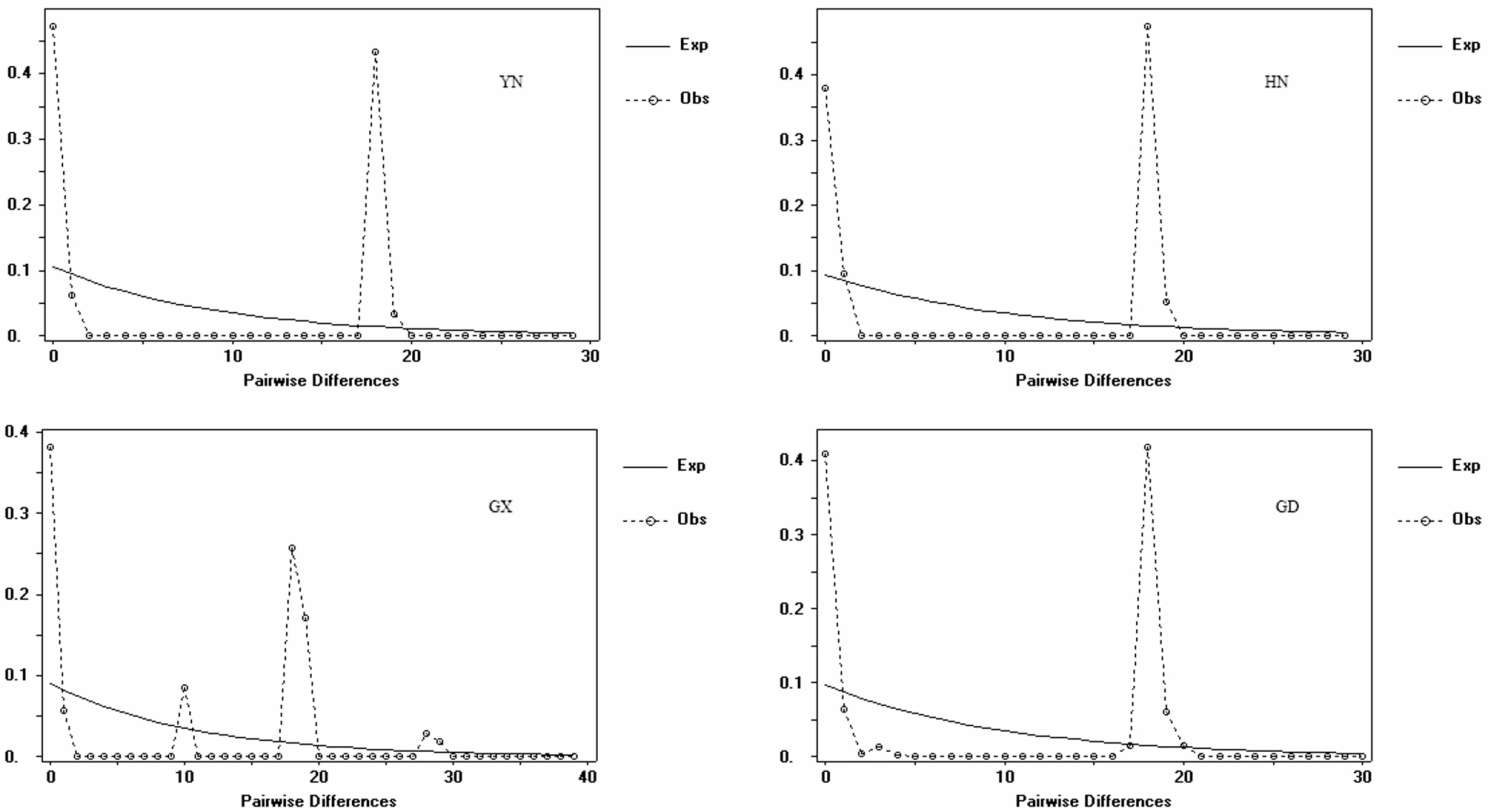
Mismatch distribution of *COI* genes of fall armyworm (FAW) populations.

**Fig. 4:**
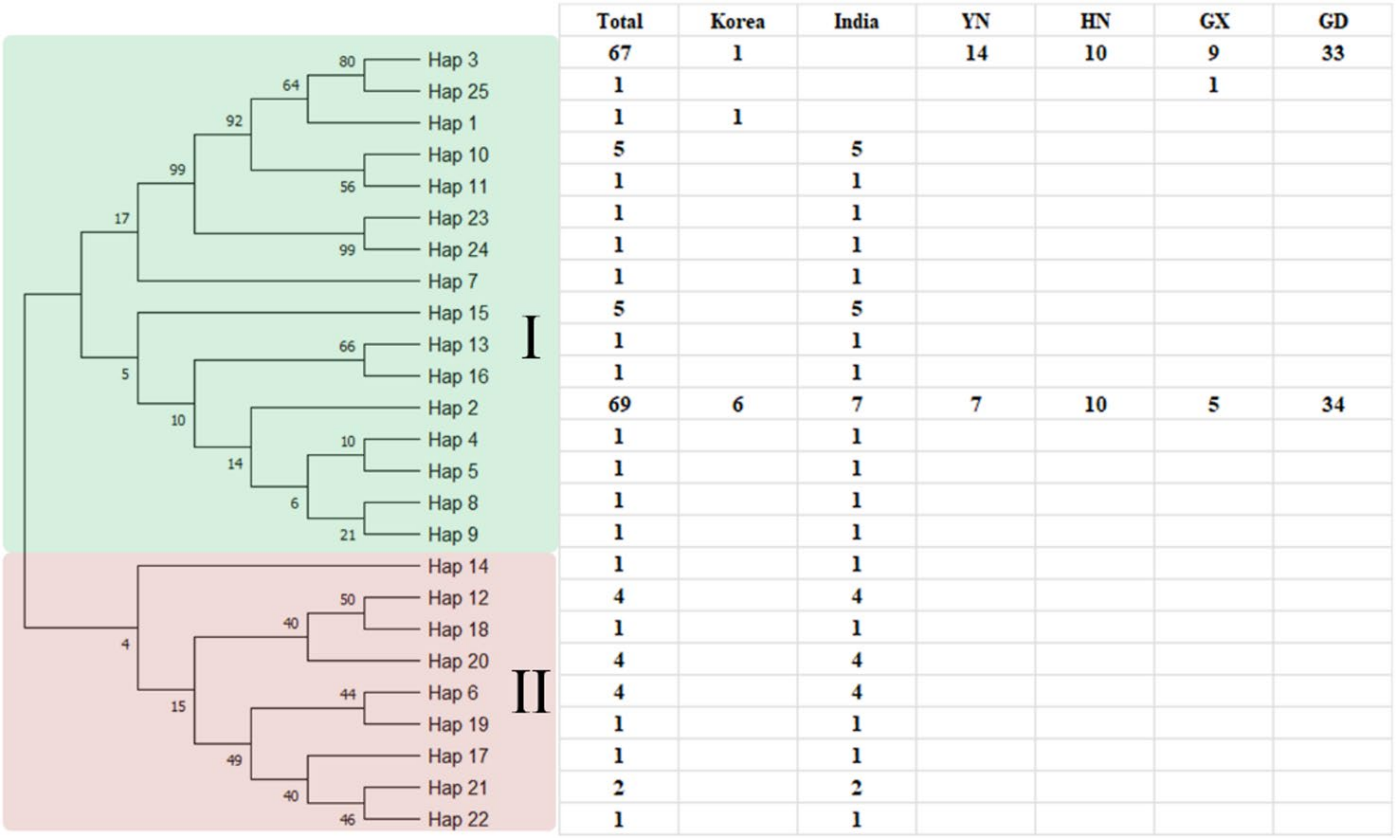
The phylogenetic of fall armyworm (FAW) haplotypes.

Indian populations displayed the highest level of genetic diversity, with 88 polymorphic sites, 95 mutations, and 22 haplotypes. Their haplotype diversity (0.942) and nucleotide diversity (0.014) were the highest among all groups. In contrast, populations from the four Chinese provinces (GD, GX, YN, HN) and South Korea exhibited markedly lower diversity, with 11–13 polymorphic sites, only 2–3 haplotypes, haplotype diversity ranging from 0.46 to 0.56, and nucleotide diversity between 0.007 and 0.009. Consequently, the overall genetic diversity of the global dataset (haplotype diversity: 0.71; nucleotide diversity: 0.01) was primarily driven by the high variation within Indian populations (Figs. 3 and 4).

Neutrality tests indicated distinct demographic histories among regions (Table 2). Indian populations showed signatures of recent population expansion or positive selection, with significant negative values for Fu’s Fs (-2.88), Fu and Li’s D* (-4.02), F* (-3.96), and Tajima’s D (-2.10). In contrast, Chinese populations (GD, YN, HN) yielded significantly positive values for Tajima’s D (GD: 3.94; YN: 2.38; HN: 3.08) and Fu and Li’s F (GD: 2.71; YN: 1.99; HN: 2.21), suggesting demographic contraction or balancing selection. Korean populations showed no significant deviation from neutrality (Tajima’s D = 0.13; Fu’s Fs = 3.86).

**Table 2:** The genetic diversity of *COI* in fall armyworm (FAW) populations from China, India, and South Korea.

	GD	GX	YN	HN	India	Korea	Total
No. of sequences	67	15	21	20	46	8	177
No. of polymorphic sites	11	13	11	11	88	11	90
No. of mutations	11	13	11	11	95	12	97
No. of haplotypes	2	3	2	2	22	3	25
Haplotype diversity	0.51	0.56	0.47	0.52	0.942	0.46	0.71
Nucleotide diversity	0.009	0.009	0.008	0.009	0.014	0.007	0.01
Fu’s Fs statistic	17.85	7.04	11.08	11.79	− 2.88	3.86	− 0.27
Fu and Li’s D test statistic	1.44	0.79	1.44*	1.44*	− 4.02**	0.95	− 7.78**
Fu and Li’s F test statistic	2.71**	1.12	1.99**	2.21**	− 3.96**	0.84	− 6.04**
Tajima’s D	3.94**	1.48	2.38*	3.08**	− 2.10*	0.13	− 1.78*

** P < 0.01; * P < 0.05. GD: Guangdong; GX: Guangxi; YN: Yunnan; HN: Hainan.

**Fig. 5:**
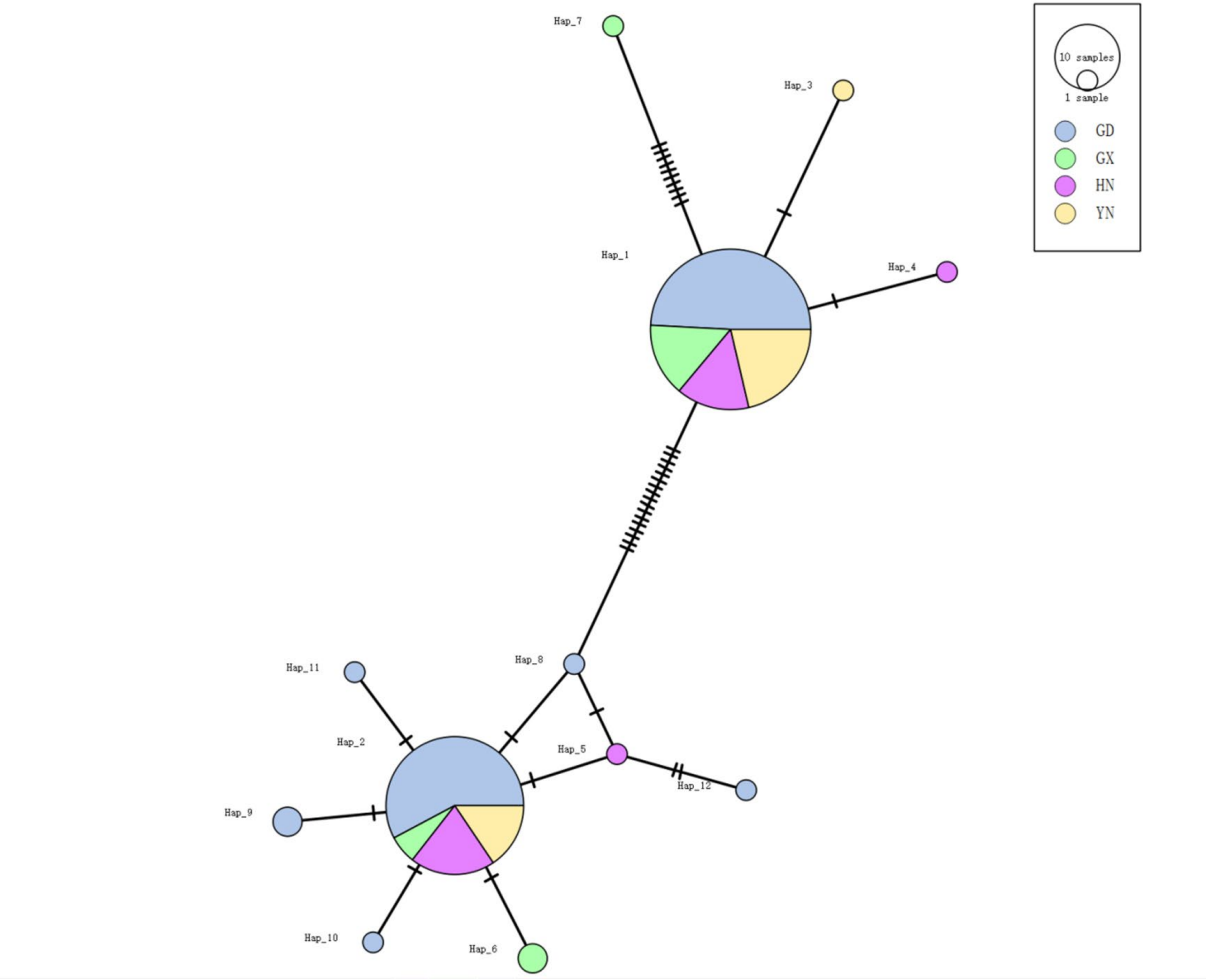
TCS network of fall armyworm (FAW) based on *COI* haplotypes.The sizes of the circles indicate the number of the individuals with each given haplotype.

A haplotype network was constructed using the TCS method to visualize relationships among the 12 haplotypes identified in Chinese populations (Fig. 5). The network displayed a star-like topology and was divided into two primary clusters: one comprising four haplotypes (Hap1, Hap3, Hap4, Hap7) and the other comprising eight (Hap2, Hap5, Hap6, Hap8–Hap12). Within this network, Hap1 occupied a central position with the highest frequency and was connected to all other haplotypes through one or few mutational steps, suggesting it may represent a foundational or ancestral haplotype for the sampled populations.

Among the 12 haplotypes, only two (Hap1 and Hap2) were shared across multiple geographic populations, while the remaining ten were private (population-specific). Hap1 was the most abundant and widespread, constituting 51.6% of all samples and being present in individuals from all four surveyed provinces in China. Hap2 was the second most frequent haplotype, accounting for 38.1% of the total samples.

### Population genetic differentiation and gene flow

To quantify the overall genetic structure, an Analysis of Molecular Variance (AMOVA) was performed on the combined dataset of six geographic regions (India, South Korea, and four Chinese provinces). The results indicated that the vast majority of genetic variation (85.37%) resided within populations, while a significant portion (14.63%) was attributable to variation among these regions (Fixation index *F*_*st*_ = 0.15, P < 0.05; Table 4).

Pairwise *F*_*st*_ and gene flow estimates provided a detailed view of inter-population relationships (Table 3). Among Chinese populations, differentiation was heterogeneous. Yunnan (YN) showed significant genetic differentiation from others (*F*_*st*_ = 0.23–0.29). In contrast, populations from Guangdong (GD) and Guangxi (GX) frequently exhibited negative *F*_*st*_ values (e.g., GD-GX: *F*_*st*_ = -0.003 to -0.033), indicating genetic similarity higher than that within subpopulations and *COI* nciding with high gene flow. Hainan (HN) populations showed moderate differentiation.

**Table 3:** Pairwise *F* < sub > ST < /sub > (below diagonal) and gene flow (*N*m, above diagonal) among 6 geographic populations of fall armyworm (FAW) based on *COI* gene.

	Korea	India	YN	HN	GX	GD
Korea	–	42.96	1.63	10.92	1.77	8.93
India	0.01	–	1.20	2.43	1.29	1.98
YN	0.23	0.29	–	69.20	inf	16.88
HN	0.04	0.17	0.007	–	inf	Inf
GX	0.22	0.27	− 0.06	− 0.003	–	24.16
GD	0.05	0.20	0.03	− 0.03	0.02	–

Inf: infinite gene flow.

In the cross-regional context, Chinese populations exhibited a range of differentiation (*F*_*st*_ = − 0.033–0.29) comparable to that observed between other regions. The analysis of gene flow revealed a distinct pattern: the highest estimated gene flow occurred between India and South Korea (Nm = 42.96), and substantial gene flow was also observed between South Korea and several Chinese populations (Nm = 8.93–10.92). This pattern suggests a potential migration corridor where India may serve as a source for South Korean populations, which in turn may act as a secondary source for populations in parts of China (Table 4).

**Table 4:** AMOVA analysis of fall armyworm (FAW) populations based on the combined dataset from six geographic regions.

Source of variation	df	Sum of squares	Variance components	Percentage of variation
Among populations	5	89.14	0.55	14.63
Within populations	171	547.51	3.20	85.37
Total	177	636.64	3.75	
Fixation index *F*_*st*_	0.15			

### Phylogenetic and haplotype network analyses

To examine the evolutionary relationships among FAW populations from different geographic regions, we constructed a neighbor-joining phylogenetic tree based on COI haplotypes from China, India, and South Korea (Fig. 4). The phylogenetic tree revealed two major clades, with Chinese haplotypes clustering predominantly within a single clade alongside some Indian and Korean haplotypes, indicating shared ancestry among these populations.

To further visualize the relationships among the 12 haplotypes identified in Chinese populations, we constructed a haplotype network using the TCS method (Fig. 5). The network displayed a star-like topology and was divided into two primary clusters: one comprising four haplotypes (Hap1, Hap3, Hap4, Hap7) and the other comprising eight (Hap2, Hap5, Hap6, Hap8–Hap12). Within this network, Hap1 occupied a central position with the highest frequency and was connected to all other haplotypes through one or few mutational steps, suggesting it may represent a foundational or ancestral haplotype for the sampled populations.

Among the 12 haplotypes, only two (Hap1 and Hap2) were shared across multiple geographic populations, while the remaining ten were private (population-specific). Hap1 was the most abundant and widespread, constituting 51.6% of all samples and being present in individuals from all four surveyed provinces in China. Hap2 was the second most frequent haplotype, accounting for 38.1% of the total samples.

## Discussion

Since its invasion into China in December 2018, the fall armyworm (*Spodoptera frugiperda*) has rapidly become a major agricultural threat. Understanding the genetic mechanisms underlying its adaptation and spread is crucial for effective management. Our study, employing both mitochondrial *COI* and nuclear *Tpi* markers, reveals distinct genetic patterns in fall armyworm (FAW) populations across China’s annual breeding area and points to their complex invasion history.

The most striking finding is the markedly lower genetic diversity in Chinese populations (haplotype diversity: 0.47–0.56; nucleotide diversity: 0.007–0.009) compared to populations from India (0.942 and 0.014, respectively). This pattern strongly supports the hypothesis that invasive populations in the Eastern Hemisphere, including those in China, originated from a limited genetic source, likely in South Asia, and experienced severe founder effects and genetic bottlenecks upon colonization^17^. The near-complete dominance of the corn-strain, as confirmed by *Tpi* genotyping in all our samples, aligns with previous reports from early invasion foci in Yunnan^15^ and global genomic analyses suggesting the American corn-strain as the primary invader of the Eastern Hemisphere^17^. This genetic homogeneity, particularly the pronounced dominance of a few haplotypes (e.g., Hap_3 in over 50% of GD and HN samples), is characteristic of recent expansion from a small number of founders. This inference is further bolstered by the significantly positive Tajima’s D values observed in Guangdong (3.94), Yunnan (2.38), and Hainan (3.08), which are indicative of population bottlenecks or balancing selection following a rapid expansion event. Notably, Guangxi (GX) exhibited relatively higher diversity, harboring all three common haplotypes. This province’s position as a potential gene flow hub, possibly receiving continuous influx from the China-Vietnam border region, may have mitigated the genetic bottleneck effects experienced elsewhere.

Population differentiation analysis revealed a complex landscape shaped by both geographic barriers and facilitating agents. A significant proportion of genetic variation (14.63%) was found among populations (*F*_*st*_ = 0.15), which is notably higher than typical for highly migratory Lepidoptera. This differentiation shows clear spatial structuring. The formidable barrier of the Himalayas is implicated in the significant genetic differentiation between Yunnan and Indian populations (*F*_*st*_ = 0.294), limiting direct migration. In contrast, within southern China, high levels of gene flow were detected. Notably, infinite gene flow estimates (Nm = ∞) among Yunnan, Hainan, and Guangxi populations, coupled with negative *F*_*st*_ values (e.g., -0.033 between GD and HN), suggest genetic fusion events. These patterns are likely driven by meteorological forces, such as typhoons and the prevailing South China Sea monsoon, which can facilitate long-distance, cross-sea migration of moths, thereby connecting populations across what would otherwise be geographic barriers. This passive wind-assisted dispersal is a key mechanism in the annual reinvasion of temperate regions by migratory pests.

The evolutionary trajectories of fall armyworm (FAW) populations, inferred from neutrality tests and diversity indices, appear to be influenced by both demographic history and local selection pressures. The signals of recent population expansion in Indian populations (significantly negative Tajima’s D and Fu’s Fs) contrast with the bottleneck signals in Chinese populations. In particular, the extremely significant positive selection signals in Guangdong populations may be linked to intense local selection pressure, potentially from widespread insecticide application. This region’s high agricultural output correlates with frequent pesticide use, which could drive the rapid fixation of resistance alleles, reducing genetic diversity at non-neutral loci. Guangxi’s non-significant neutrality values may reflect its role as a genetic mixing zone, where continuous gene flow dilutes localized selection signatures. These demographic inferences are consistent with field survey data showing fluctuating, low-density populations in southern overwintering areas (e.g., 1–2 larvae per 100 plants in Hainan), which are prone to genetic drift.

In conclusion, our genetic analysis depicts an invasion scenario for fall armyworm (FAW) in China characterized by a founding event from a limited South Asian source, followed by rapid population expansion that led to reduced genetic diversity. Subsequent population dynamics have been shaped by a combination of geographic barriers (e.g., the Himalayas), facilitating weather patterns (monsoons), and potentially human-induced selection (insecticide use). The identification of Guangxi as a high-diversity zone and gene flow conduit underscores the importance of cross-border biosecurity and regional monitoring. Future management strategies should consider these genetic insights, emphasizing area-wide integrated pest management to mitigate resistance evolution and monitoring migratory corridors to predict and intercept population inflows.

## Supplementary Information

Below is the link to the electronic supplementary material.

Supplementary Material 1

Supplementary Material 2

Supplementary Material 3

Supplementary Material 4

Supplementary Material 5

Supplementary Material 6

Supplementary Material 7

Supplementary Material 8

Supplementary Material 9

Supplementary Material 10

## Data Availability

Sequence data that support the findings of this study have been deposited in the National Center for Biotechnology Information with the primary accession code PQ129852-PQ129947,PQ057544-PQ057548,PQ056730-PQ056734,PQ129286-PQ129291,PQ129316-PQ129321,PQ129486-PQ129490.
