# Supplementary material for: Population genetic diversity in the annual breeding area of the Spodoptera frugiperda in China
Source: Sci Rep. 2026 Apr 2;16:15826. doi: 10.1038/s41598-026-46482-1 (PMC13194687; doi:10.1038/s41598-026-46482-1)
Supplement: Supplementary file 10 — Supplementary Material 10 [file 41598_2026_46482_MOESM10_ESM.docx]

Table 1. FAW samples collection information.

| Geographicregion | Sampling locations | Sampling Code | Sampling date | No.of individual | Longitude Latitude | Accession numbers |
| --- | --- | --- | --- | --- | --- | --- |
| Guangxi(GX)  Guangxi(GX)  Guangxi(GX) | Nanning | GXNN | 2023-03-15 | 5 | 108.23°E,22.61°N | PQ129486-PQ129490 |
|  | Qinzhou | GXQZ | 2023-03-16 | 5 | 108.59°E,21.93°N | PQ129908-PQ129912 |
|  | Baise | GXBS | 2023-03-17 | 5 | 106.72°E,23.59°N | PQ129897-PQ129901 |
| Yunnan(YN)  Yunnan(YN)  Yunnan(YN) | Yuxi | YNYX | 2023-03-18 | 5 | 102.57°E,24.29°N | PQ129943-PQ129947 |
|  | Puer | YNPE | 2023-03-19 | 6 | 101.05°E,23.05°N | PQ129921-PQ129926 |
|  | Xishuangbanna | YNXS | 2023-03-19 | 10 | 100.77°E,21.98°N | PQ129927-PQ129936 |
| Guangdong(GD)  Guangdong(GD)  Guangdong(GD)  Guangdong(GD)  Guangdong(GD)  Guangdong(GD)  Guangdong(GD)  Guangdong(GD)  Guangdong(GD)  Guangdong(GD)  Guangdong(GD)  Guangdong(GD) | Potou,Zhanjiang | GDZJ1 | 2023-03-04 | 5 | 110.46°E,21.24°N | PQ057544-PQ57548 |
|  | Leizhou,Zhanjiang | GDZJ2 | 2023-02-22 | 6 | 110.10°E,20.91°N | PQ129857-PQ129862 |
|  | Xuwen,Zhanjiang | GDZJ3 | 2023-02-22 | 6 | 110.18°E,20.33°N | PQ129316-PQ129321 |
|  | Suixi,Zhanjiang | GDZJ4 | 2023-02-23 | 6 | 110.25°E,21.38°N | PQ129869-PQ129874 |
|  | Lianjiang,Zhanjiang | GDZJ5 | 2023-02-23 | 6 | 110.29°E,21.61°N | PQ129863-PQ129868 |
|  | Kaifaqu,Zhanjiang | GDZJ6 | 2023-02-21 | 5 | 110.16°E,21.65°N | PQ129875-PQ129879 |
|  | Wuchuan,Zhanjiang | GDZJ7 | 2023-02-23 | 6 | 110.78°E,21.44°N | PQ129880-PQ129885 |
|  | Xiashan,Zhanjiang | GDZJ8 | 2023-02-21 | 5 | 110.40°E,21.19°N | PQ129886-PQ129890 |
|  | Mazhang,Zhanjiang | GDZJ9 | 2023-03-14 | 6 | 110.33°E,21.26°N | PQ129891-PQ129896 |
|  | Huazhou,Maoming | GDMM | 2023-03-02 | 6 | 110.64°E,21.66°N | PQ129286-PQ129291 |
|  | Shenzhen | GDSZ | 2023-03-04 | 5 | 114.06°E,22.54°N | PQ129852-PQ129856 |
|  | Guangzhou | GDGZ | 2023-03-04 | 5 | 113.26°E,23.13°N | PQ056730-PQ056734 |
| Hainan(HN)  Hainan(HN)  Hainan(HN) | Sanya | HNSY | 2023-03-03 | 6 | 109.51°E,18.25°N | PQ129937-PQ129942 |
|  | Dongfang | HNDF | 2023-03-02 | 6 | 108.65°E,19.10°N | PQ129902-PQ129907 |
|  | Haikou | HNHK | 2023-04-08 | 8 | 110.20°E,20.05°N | PQ129913-PQ129920 |

Table 2. The genetic diversity of COI in FAW populations in 4 provinces of China.

|  | GD | GX | YN | HN | India | Korea | Total |
| --- | --- | --- | --- | --- | --- | --- | --- |
| No. of sequences | 67 | 15 | 21 | 20 | 46 | 8 | 177 |
| No. of sites | 646 | 646 | 646 | 646 | 646 | 646 | 464 |
| No. of polymorphic sites | 11 | 13 | 11 | 11 | 88 | 11 | 90 |
| No. of mutations | 11 | 13 | 11 | 11 | 95 | 12 | 97 |
| No. of haplotypes | 2 | 3 | 2 | 2 | 22 | 3 | 25 |
| Haplotype diversity | 0.51 | 0.56 | 0.47 | 0.52 | 0.942 | 0.46 | 0.71 |
| Nucleotide diversity | 0.009 | 0.009 | 0.008 | 0.009 | 0.014 | 0.007 | 0.01 |
| Fu’s Fs statistic | 17.85 | 7.04 | 11.08 | 11.79 | -2.88 | 3.86 | -0.27 |
| Fu and Li’s D* test statistic | 1.44 | 0.79 | 1.44* | 1.44* | -4.02** | 0.95 | -7.78** |
| Fu and Li’s F* test statistic | 2.71** | 1.12 | 1.99** | 2.21** | -3.96** | 0.84 | -6.04** |
| Tajima’s D | 3.94** | 1.48 | 2.38* | 3.08** | -2.10* | 0.13 | -1.78* |

**Note:** **, P < 0.01; *, P < 0.05.

Table 3. AMOVA analysis of the FAW population across 4 provinces in China.

| Source of variation | df | Sum of squares | Variance components | Percentage of variation |
| --- | --- | --- | --- | --- |
| Among populations | 5 | 89.14 | 0.55 | 14.63 |
| Within populations | 171 | 547.51 | 3.20 | 85.37 |
| Total | 177 | 636.64 | 3.75 |  |
| Fixation index FST | 0.15 |  | |  |

Table 4. Pairwise Fst (in the lower left) and gene flow (in the upper right) among 4 geographic populations of FAW based on COI gene.

|  | Korea | India | YN | HN | GX | GD |
| --- | --- | --- | --- | --- | --- | --- |
| Korea | - | 42.96 | 1.63 | 10.92 | 1.77 | 8.93 |
| India | 0.01 | - | 1.20 | 2.43 | 1.29 | 1.98 |
| YN | 0.23 | 0.29 | - | 69.20 | inf | 16.88 |
| HN | 0.04 | 0.17 | 0.007 | - | inf | Inf |
| GX | 0.22 | 0.27 | -0.06 | -0.003 | - | 24.16 |
| GD | 0.05 | 0.20 | 0.03 | -0.03 | 0.02 | - |

Inf：infinitely


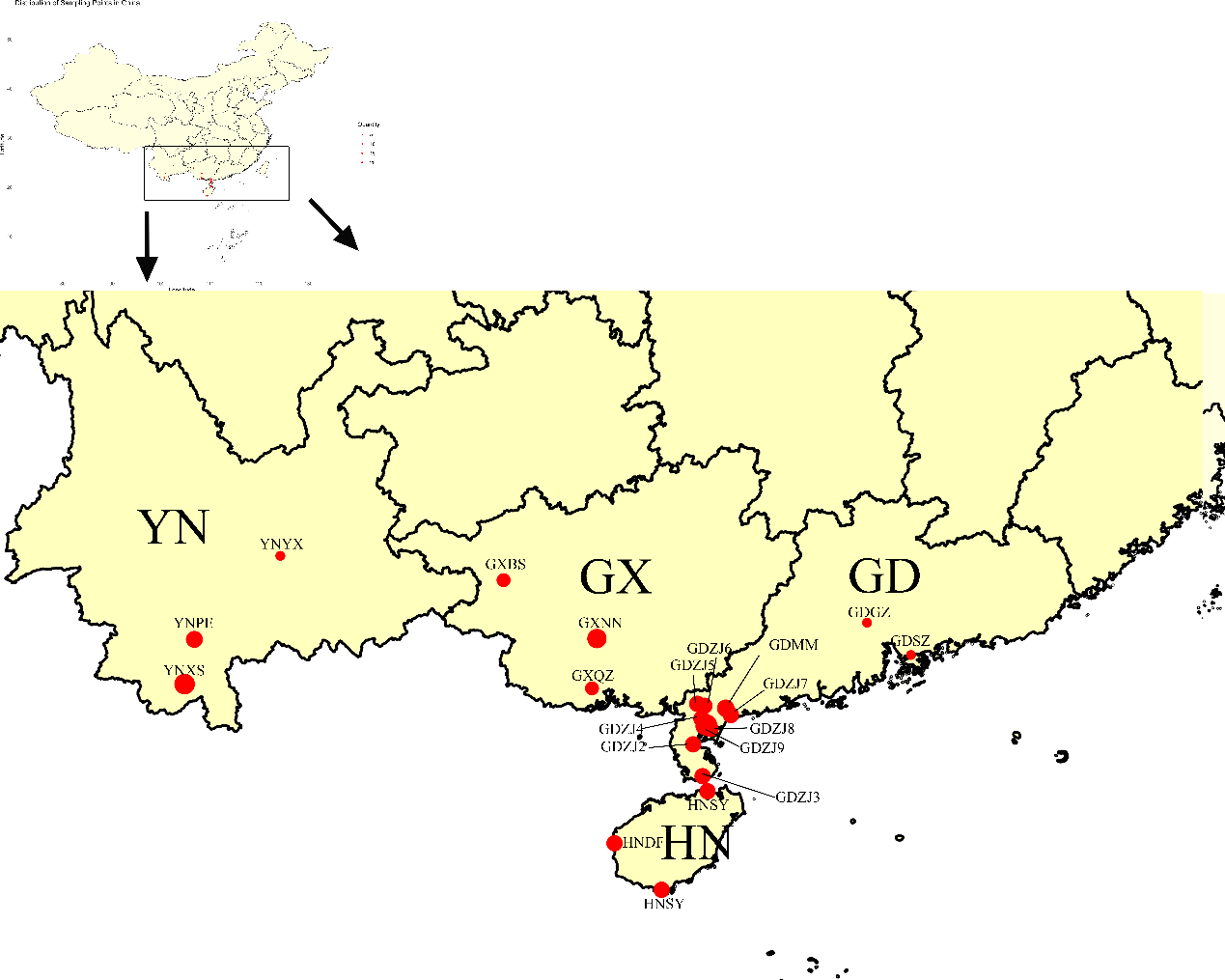


Figure 1. Sampling locations of FAW populations in China. Solid red circles are sampling locations for this study.

**Note**: The sampling map was created using:

R programming language (v4.3.2; https://www.r-project.org/)

sf package (v1.0-16; https://r-spatial.github.io/sf/) for spatial data processing

ggplot2 package (v3.5.0; https://ggplot2.tidyverse.org) for visualization

GDAL library (v3.8.2; https://gdal.org) as underlying spatial engine


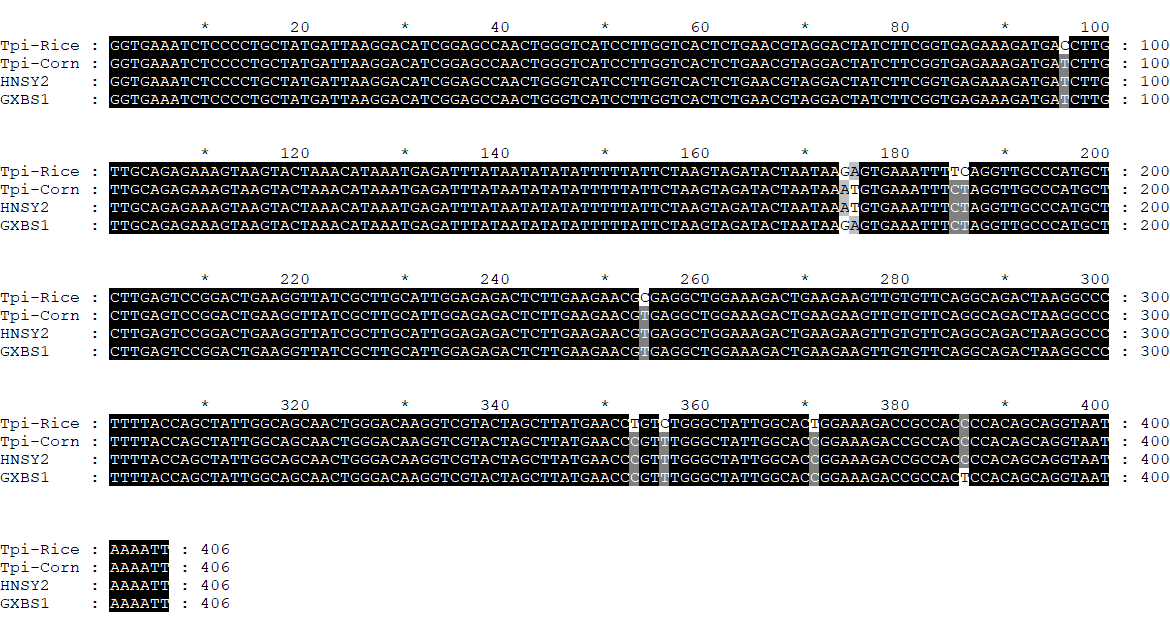


Figure 2. Identification of subtypes of S. frugiperda based on Tpi gene fragment


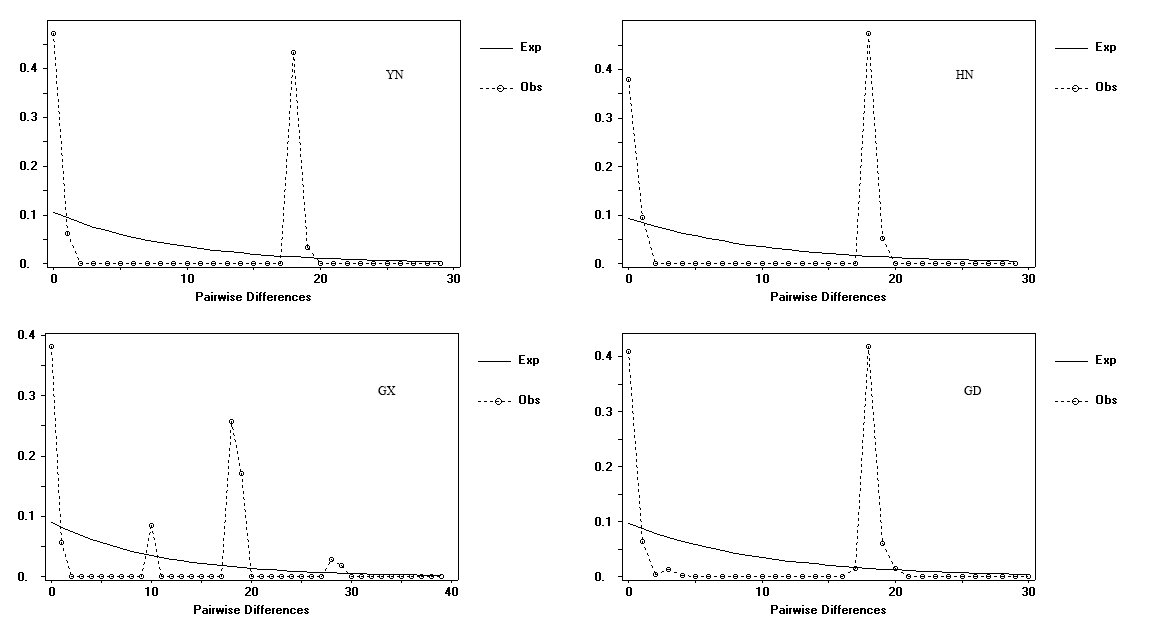


Figure 3. Mismatch distribution of COI genes of FAW populations.


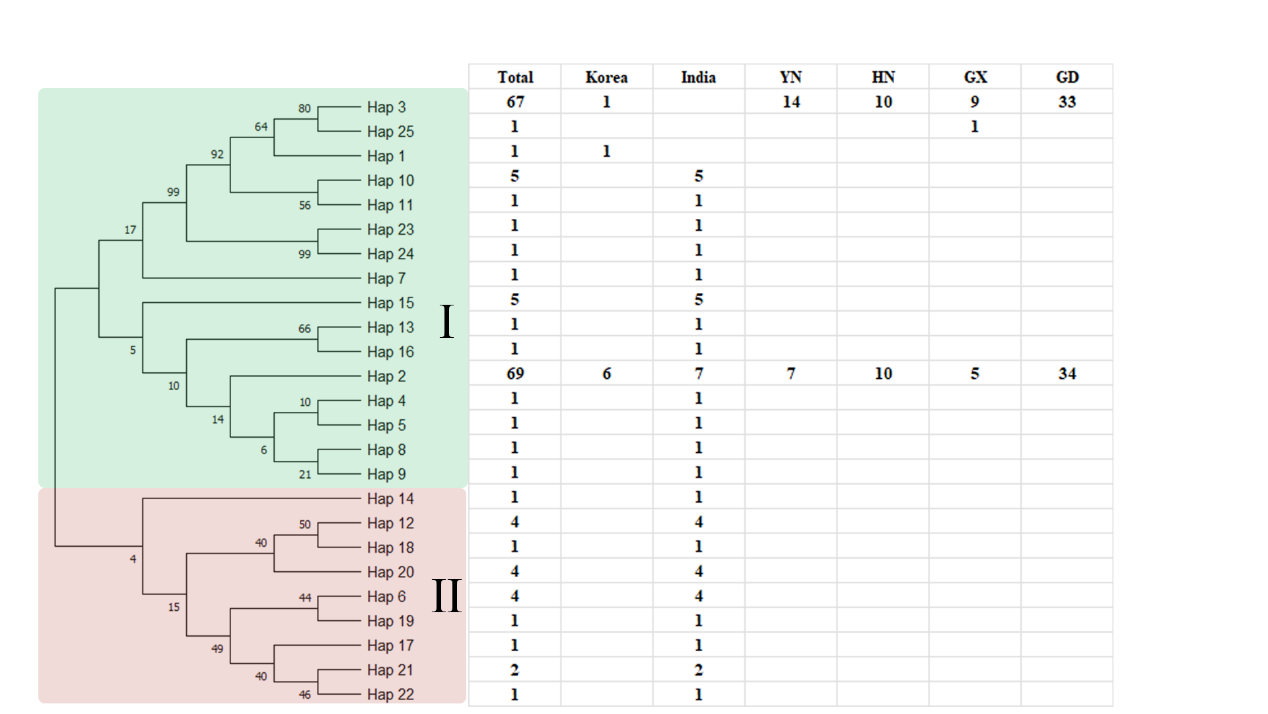


Figure 4. The phylogenetic of FAW haplotypes. The distribution and number of haplotypes in different populations are shown in the right panel.


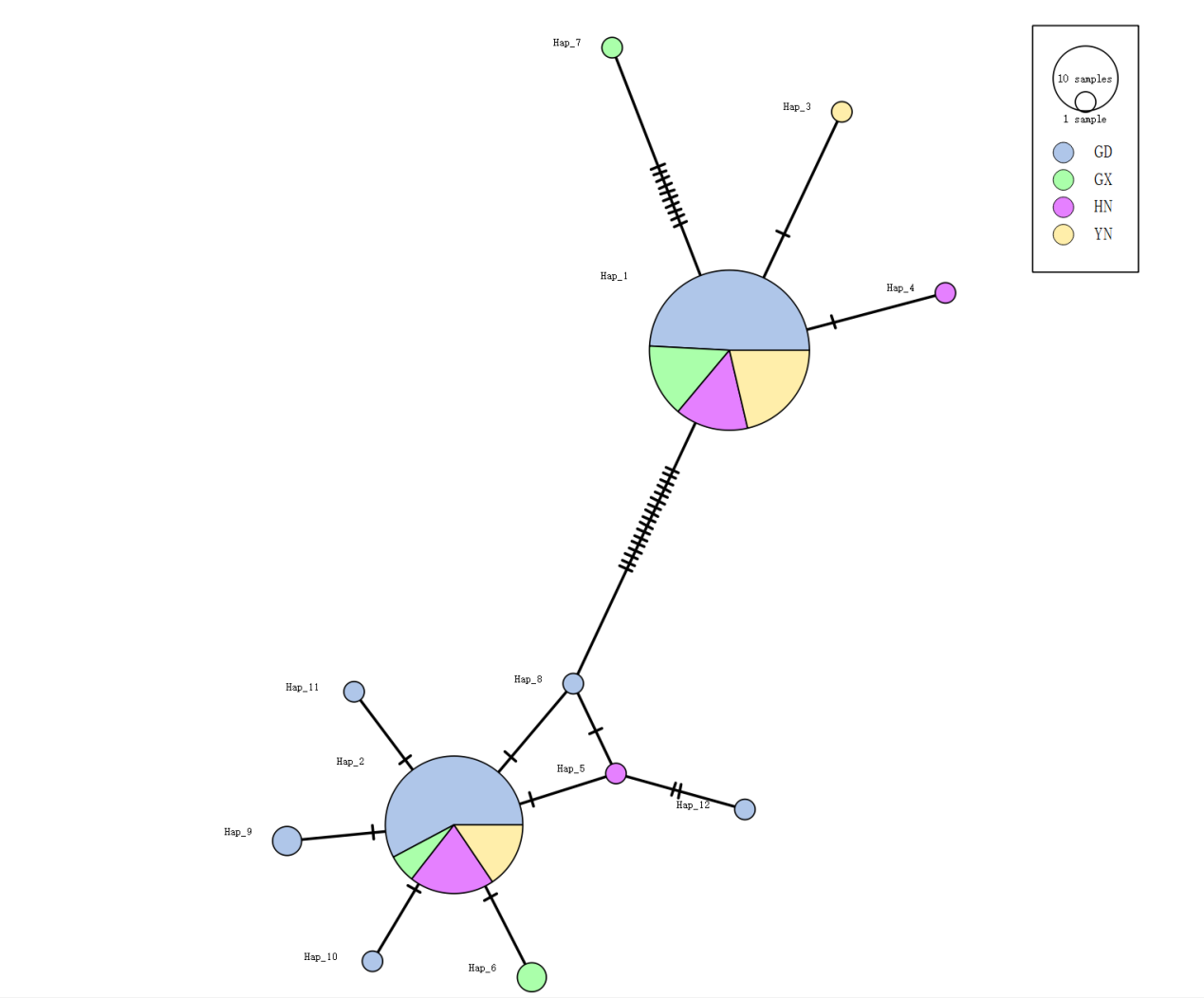


Figure 5. TCS network of FAW based on *COI* haplotypes.The sizes of the circles indicate the number of the individuals with each given haplotype.
